# Crystalized Sub-Acetate of Lead, and Tincture of Digitalis in Hypertrophy of the Heart

**Published:** 1856-11

**Authors:** W. F. Jones

**Affiliations:** Petersburg, Va.


*Crystalized Sub-Acetate of Lead, and Tincture of Digitalis in Hypertrophy of the Heart.* Reported by W. F. Jones, M.D. Petersburg, Va.

During the month of February, 1856, I was called to see Mrs. J. æt. 57. Has been, for the past five or six years, subject to frequent attacks of rheumatism—first showing itself in the hand and wrist, and afterwards becoming general. Found her, on my first visit, just recovering from a violent paroxysm of palpitation of the heart, accompanied by distressing dyspnœa; the chest heaving with laborious breathing; pulse full and hard; countenance rather pallid; tongue slightly furred and pale; appetite good. I bled her to about sixteen ounces. The relief was prompt. Ordered digitalis combined with assafœtida. There was no return of the above symptoms for some three or four weeks.

Upon the next attack I again bled her, taking about six ounces, but without any apparent benefit. Upon placing the ear over the region of the heart, found its action forcible and labored; pulse full, strong, hard, and frequent, but regular; no intermission in its pulsations were at any time perceived. Ordered cups to the cardiac region, also between the shoulders, followed by the external use of Croton oil; bowels to be kept in a soluble state by the use of mild laxatives.

From this period a variety of prescriptions were used, including some of the preparations of iron, but without any permanently good results. Indeed, my patient was getting worse.

*May 7th.*—Up to this period Mrs. J. has suffered from more frequent attacks of palpitation, dyspnœa, &c. and her sufferings are very great. Having satisfied my mind as to the character of the disease, I had determined to try the effects of a combination of the sub-acetate of lead with digitalis, as recommended by J. L. Brachet. I accordingly ordered it to be taken in doses of one grain of the sub-acetate with ten drops of digitalis, three times a-day. It may be proper to remark here, that my patient can lie down with ease and comfort at any time when not under the influence of these paroxysms; but, upon making any considerable effort, an attack is brought on.

*May 8th.*—On my visit to-day, Mrs. J. informed me that she thought herself better; had passed a quiet night; attacks less frequent, and of shorter duration; she also complains of less distress during the paroxysm.

*May 9th.*—Rather more comfortable to-day; has had no return of paroxysm; appetite not good; did not rest well last night. Continued same treatment.

*May 10th.*—No return of paroxysm; pulse decidedly improved. I think she is now under the influence of the digitalis and acet. plumb. Made no change in my treatment.

*May 11th.*—Mrs. J. considers herself much better to-day; no return of her attacks. Treatment continued.

*May 12th.*—Decidedly improved in every particular; no indication of a tendency to return of former distressing symptoms.

My patient now proposed a visit to the country. I therefore directed her to pursue the same course; which was done, and with the benefit of keeping off her former attacks. She has returned now, the 10th August, and does not complain of any of her former symptoms. She, however, looks somewhat debilitated at present, from a slight bilious attack while absent in the country, but every vestige of the cardiac affection is removed, and at this time attends to her domestic duties.

*Diagnosis.*—I suppose the above case to be one of well-developed hypertrophy of the heart, dependant, in the first instance, upon rheumatic neuralgia, translated to the cardiac region. The treatment, which seemed so efficacious and prompt in its results, was suggested to my mind by an article first published in the *New Orleans Medical News and Hospital Gazette*, under the head of "Observations on the Use of Crystalized Sub-Acetate of Lead in Hypertrophy of the Heart," by J. L. Brachet. Although my prescription differs somewhat from his, still it amounts to the same thing, and to him I willingly concede the credit due him.

*Modus Operandi.*—The digitalis is known to exert a *sedative* influence on the heart, diminishing the force and frequency of its action, while the acet. plumb. has a *sedative astringent* effect, thereby aiding the digitalis in its efforts to command the excessive action of the heart, while it contracts its fibres. This contraction produces pressure, which pressure arouses the absorbents, and the difficulty is removed. This, however, is but theory—facts are what we want.

The above case was certainly most promptly relieved by the continued agency of the above remedies. I report it for the benefit of my professional brethren. I take no credit to myself for any discovery. I have simply tested the recommendation of another, and give you the result.—*Virginia Medical Jour.*

